# Ghrelin Enhancer, the Latest Evidence of Rikkunshito

**DOI:** 10.3389/fnut.2021.761631

**Published:** 2021-12-09

**Authors:** Chihiro Yamada, Tomohisa Hattori, Shunsuke Ohnishi, Hiroshi Takeda

**Affiliations:** ^1^Tsumura Kampo Research Laboratories, Tsumura & Co., Ibaraki, Japan; ^2^Laboratory of Molecular and Cellular Medicine, Faculty of Pharmaceutical Sciences, Hokkaido University, Sapporo, Japan; ^3^Gastroenterology, Tokeidai Memorial Hospital, Sapporo, Japan; ^4^Faculty of Pharmaceutical Sciences, Hokkaido University, Sapporo, Japan

**Keywords:** ghrelin, GHS-R, anorexia, rikkunshito, Kampo, stress, aging

## Abstract

Rikkunshito is a Japanese herbal medicine (Kampo) that has been attracting attention and researched by many researchers not only in Japan but also worldwide. There are 214 rikkunshito articles that can be searched on PubMed by August 2021. The reason why rikkunshito has attracted so much attention is due to an epoch-making report (Gastroenterology, 2008) discovered that rikkunshito promotes the secretion of the orexigenic peptide ghrelin. Since then, many researchers have discovered that rikkunshito has a direct effect on the ghrelin receptor, GHS-R1a, and an effect of enhancing the ghrelin signal to the brain. Additionally, a lot of evidence that rikkunshito is expected to be effective for various gastrointestinal diseases have also been demonstrated. Numerous basic and clinical studies have suggested that rikkunshito affects (i) various discomforts caused by anticancer drugs, gastroesophageal reflux disease, functional dyspepsia, (ii) various stress-induced anorexia, (iii) hypophagia in the elderly, and (iv) healthy lifespan. In this review, as one who discovered the ghrelin enhancer effect of rikkunshito, we will review the research of rikkunshito so far and report on the latest research results.

## Rikkunshito

Rikkunshito is one of the prescriptions described in the old medical book *Return of Spring from All Kinds of Diseases* compiled by Kyoenken in 1587. Rikkunshito comprises eight herbal medicines, *Atractylodis lanceae rhizoma, Ginseng radix, Pinelliae tuber, Hoelen, Zizyphi fructus, Aurantii nobilis pericarpium, Glycyrrhizae radix* and *Zingiberis rhizoma*. It was used for patients with gastrointestinal weakness, loss of appetite, epigastrium, tiredness, anemia, and chills in the limbs. In Japan, it is an insurance coverage drug for gastritis, gastric atony, gastroptosis, indigestion, loss of appetite, stomach pain, and vomiting, which is covered by a doctor's prescription. The first high-quality evidence is a multicenter comparative study of TJ-43 rikkunshito for indefinite gastrointestinal complaints, such as chronic gastritis by Harasawa et al. (1). Additionally, a multicenter, double-blind study using rikkunshito has recently been conducted, including proton pump inhibitor refractory non-erosive reflux disease (NERD, *n* = 242) (2) and functional dyspepsia (*n* = 192) (3), and it was proven to be effective for them. Rikkunshito was compared to the placebo treatment group, and the degree of improvement of total and the acid-related dysmotility symptom scores of the frequency scale for the symptoms of gastroesophageal reflux disease (FSSG) after the 8-week treatment was significantly greater in the rikkunshito group than in the placebo group. Rikkunshito also significantly increased the global assessment of overall treatment efficacy in functional dyspepsia patients and improved upper gastrointestinal symptoms after 8 weeks, especially postprandial fullness/early satiety and bloating. Thus, except acid secretion inhibitors and acotiamide, there is poor evidence of drugs for digestive system complaints so far, so the accumulation of clear evidence by Japanese Kampo medicine is an important event.

Additionally, rikkunshito's mechanisms of action have been extensively studied in detail. Rikkunshito has been proven to enhance gastric emptying (4–6) and promote adaptive relaxation reaction (6–10) of the stomach from basic and clinical aspects. These actions are not merely single pharmacological actions, such as increased gastric motility or decreased gastric acid secretion, but have the characteristic of enhancing the overall function of the stomach. Thus, it can be said that the mechanism of action of rikkunshito is significantly different from that of new drugs with a single pharmacological action.

Furthermore, the epoch-making evidence that rikkunshito was made known to global gastroenterologists was believed to be discovering the ghrelin-enhancing effect of the orexigenic peptide by rikkunshito (11). Inspired by the common characteristics of patients after taking rikkunshito, we discovered that rikkunshito may enhance the action of ghrelin and proved the first evidence. After that, many researchers proceeded with further detailed research, and rikkunshito enhanced the gene expression of the ghrelin receptor GHS-R1a (12) and that the signal transduction of ghrelin was enhanced by improving the binding between ghrelin and GHS-R1a (13).

This review focuses on the effects of rikkunshito on ghrelin and describes the latest evidence and the following possibilities regarding the potential treatment of rikkunshito for various diseases.

## Action and Mechanism on Ghrelin

### What Is Ghrelin?

**Figure 1:**
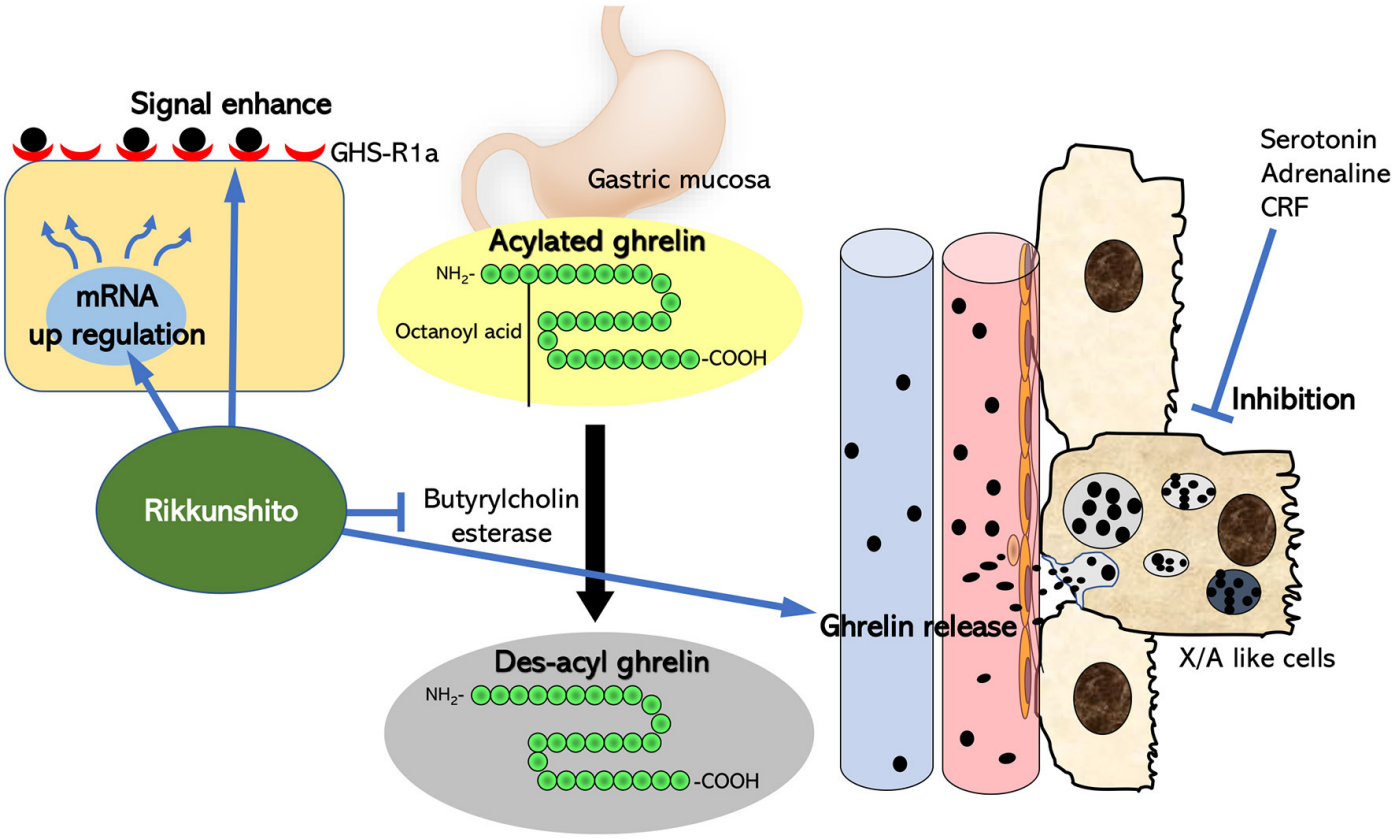
Summary of the mechanism of action of rikkunshito as a ghrelin enhancer. Ghrelin is produced from X/A-like cells near the gastric mucosa, and the ghrelin receptors at the ends of the vagus nerve in the surrounding tissues are stimulated. Most of it migrates to blood vessels and is carried to the brain. Serotonin, adrenaline, and CRF negatively regulate ghrelin secretion in the periphery. Rikkunshito suppresses the decrease in ghrelin that occurs in diseases by antagonistically acting on these factors with receptors in the periphery or central nervous system. Furthermore, rikkunshito enhances the binding of ghrelin to the ghrelin receptor and also acts on the ghrelin signal transduction system itself to enhance the ghrelin signal.

Ghrelin is a unique peptide with 28 amino acids and an n-octanoyl group. Its main production site is localized to X/A-like cells in the gastric mucosa (14). The hunger signal from periphery is increased by the production of ghrelin, for example, hypoglycemia. As a reflection, it is also increased in the blood. The orexigenic activity of ghrelin is triggered by the serine 3-acyl modification of octanoic acid (acylated ghrelin). This acylation process is catalyzed by the gastric membrane-binding protein, ghrelin *O*-acyltransferase (GOAT) (15). However, acylated ghrelin released into tissues or blood is immediately metabolized by liver-derived butyrylcholinesterase (16) to des-acyl ghrelin, which has no orexigenic effect (Figure 1). A difficult point in measuring the active form of acylated ghrelin in clinical research is the instability of acylated ghrelin. The half-life after intravenous injection of acylated ghrelin is 8 min in rats (17). In the case of animal experiments, it should be conducted within 3–5 min from blood sampling to centrifugation by multiple researchers before adding concentrated hydrochloric acid and freezing in liquid nitrogen. A deep freezer at −80°C is optimal for storage. However, it is not easy to freeze within this time after collecting blood from a patient. Therefore, if the conditions at the time of blood collection are different, it is expected that the acylated ghrelin value will also differ significantly from facility to facility or from day to day.

The target of the ghrelin ligand is growth hormone secretagogue receptor 1a (GHS-R1a), a 7-transmembrane G protein-coupled receptor composed of 366 amino acid residues. GHS-R1a is present in the terminal of the vagal afferent nerve, pancreatic cells, spleen, myocardium, bone, fat, thyroid gland, adrenal gland, and immune cells (18, 19). Particularly, it is also densely expressed in the hypothalamic nucleus in the central nervous system (18, 19). The peripheral to the central ghrelin signal is initiated by the binding of GHS-R1a present at the vagal nerve terminal in the gastric mucosa to acylated ghrelin, and the signal to the central nervous system is transmitted. Ghrelin bound to GHS-R1a transmits its starvation signal via the solitary nucleus (NTS) of the medulla oblongata to neuropeptide Y (NPY)/Agouti-related peptide (AgRP) neurons, which are localized in the arcuate nucleus of the hypothalamus and the signal is transmitted to higher centers. Ghrelin stimulates NPY/AgRP neurons and promotes the production of NPY/AgRP peptides and proceeds to induce appetite (20) and promotes gastrointestinal fasting contraction via the vagus nerve efferent pathway (21, 22). GHS-R1a is also localized in the ventral tegmental area (23) and hippocampus (24). Thus, in addition to the action on appetite and energy metabolism, ghrelin may also be involved in cognition and memory and is thought to play an essential role in maintaining neuropsychiatric homeostasis in both peripheral and central tissues.

Serotonin and the adrenaline system play a major role in energy metabolism and regulate the secretion of ghrelin-producing cells, X/A-like cells in the gastric mucosa (11, 25–27). Particularly, intraperitoneal (IP) administration of an agonist of serotonin 2B receptor (5-HT_2B_R) BW723C86 or an agonist of 5-HT_2C_R, meta-chlorophenyl piperazine (mCPP), to rats significantly reduces acylated ghrelin levels in peripheral blood (11, 25). Administration of the 5-HT_2B_R or _2C_R antagonists significantly reversed decreases in peripheral ghrelin and food intake in several disease models (11, 28–30). Cell damage releases large amounts of serotonin after chemotherapy such as cisplatin administration, but it is easy to imagine activating 5-HT_2B_R and 5-HT_2C_R present in the gastric smooth muscle and brain. Administering these receptor antagonists to cisplatin-treated rats simultaneously restores reduced food intake and peripheral acylated ghrelin (11).

Acute stress increases the secretion of adrenaline and activates various receptors. Clonidine, an adrenergic α2 receptor agonist, also lowered peripheral acylated ghrelin concentration. Conversely, the addition of noradrenaline or adrenaline to the ghrelinoma cell lines stimulated ghrelin secretion, and this effect was blocked by atenolol (31), a selective β1-adrenergic antagonist. Systemic administration of isoproterenol and denopamine restores reduced blood acylated ghrelin levels in stress models. In a stress-like model with intracerebroventricular (ICV) urocortin injection, the administration of adrenergic α-receptor antagonist phentolamine and the α2 receptor antagonist yohimbine significantly improved the decreased acylated levels (32). The effect disappeared when administered along with a ghrelin receptor antagonist. This means that adrenergic α2 receptor stimulation negatively regulated feeding through a decrease in ghrelin receptor activation. ICV administration of the corticotropin-releasing factor (CRF), a trigger factor for the HPA axis during stress, also reduces peripheral blood acylated ghrelin levels and food intake (29). Thus, abnormal acylated ghrelin secretion may be noticed in diseases in which these factors are closely related to pathology.

### Promotion of Ghrelin Secretion by Rikkunshito

#### Normal Animals and Healthy Volunteers

A few reports examine the effects of the administration of rikkunshito on normal mice to increase appetite and acylated ghrelin production. Matsumura et al. measured blood acylated ghrelin concentration and gastric gene expression at 0.7–1.4% drinking water administration of rikkunshito to normal mice and showed a significant increase in the rikkunshito group compared with the saline-treated group (33). However, in our preliminary study, the amount of acylated ghrelin in the blood tended to increase 180 min after the administration of rikkunshito 1 g/kg by gavage (distilled water administration group, 55.7 ± 9.2 fmol/mL, vs. rikkunshito administration group, 72.3 ± 5.9 fmol/mL), and des-acyl concentration decreased (distilled water administration group, 729.6 ± 74.1 fmol/mL, vs. rikkunshito administration group, 407.1 ± 30 fmol/mL). It is considered that the difference in this result depends on the difference in administration period and administration method. Additionally, Matsumura et al. demonstrated that administration of rikkunshito 7.5 g/day to 21 healthy volunteers for 2 weeks significantly increased blood acylated ghrelin levels compared to that before administration (33). It is considered that the maintenance of physiological homeostasis related to the secretion of appetite-related hormones is strictly controlled. Therefore, the effect of a single dose of rikkunshito on healthy subjects may be limited. To affect normal ghrelin secretion function, rikkunshito may require continuous administration.

### Side Effects of Chemotherapy and Cancer Cachexia

#### Animal Model

Table 1 summarizes the major indexes and ghrelin-promoting effects of rikkunshito on anticancer drug administration, organ removal, and cancer cachexia. Administering cisplatin to rodents results in reduced food intake. Peritoneal administration of cisplatin at 2 mg/kg significantly reduces rat feeding under fasting and free fed conditions up to 24 h. Gavage oral administration at a dose of 1 g/kg of rikkunshito was observed to significantly suppress the reduction in food intake due to cisplatin administration compared to saline-administered rats (11–13, 34, 35). Intraperitoneal administration of cisplatin 2 mg/kg decreases food intake and decreases blood acylated ghrelin levels and hypothalamic acylated ghrelin release 2 h after administration. Rikkunshito has been proven to abolish these declines (11, 12, 35). Similarly, rikkunshito suppressed the decrease in food intake in rats given intraperitoneally at a higher dose of cisplatin (6 mg/kg) (36). Thus, the effect of rikkunshito on ghrelin is very reproducible, and it has been proven that rikkunshito inhibits feeding reduction and an acylated ghrelin secretion-promoting effect in cisplatin-administered rats. Moreover, in cancer-bearing animals, experimental exposure to cancer cells induces cachexia, depending on the type of cancer and the duration of the experiment, resulting in a marked decrease in food intake and body weight. Additionally, a significant reduction in food intake and body weight is also observed in gastrectomized rats (37). An extreme decline in feeding leads to increased starvation and induction of signal abnormalities in peripheral appetite-promoting peptides, despite the lack of appetite in these animal models. Particularly, peripheral acylated ghrelin increases, and exogenous acylated ghrelin reactivity also decreases the so-called ghrelin resistance (13, 38). Rikkunshito administration to cancer cachexia rats is shown to improve the decrease in food intake and prolong life. However, rikkunshito did not further increase blood acylated ghrelin. Rikkunshito not only increases peripheral ghrelin but also stimulates ghrelin receptor signaling and stimulates feeding (13, 38). This mechanism of action of rikkunshito will be focused on in detail at the bottom.

**Table 1:** The effect of rikkunshito on the main evaluation and on ghrelin in each study.

**Years**	**Basic research**	**Ghrelin**	**Index**	**References**
2008	Cisplatin-treated rats	↑	Food intake↑	Takeda et al. (11)
2010	Cisplatin-treated rats	↑	Food intake↑	Yakabi et al. (12)
2011	Cancer cachexia rats	↑	Food intake, survival↑	Fujitsuka et al. (13)
2011	Cisplatin-treated rats	↑	Food intake↑	Sadakane et al. (35)
2013	Cisplatin-treated rats	-	Food intake↑	Yoshimura et al. (36)
2016	Gastrectomied rats	→	Food intake↑	Taguchi et al. (37)
2017	Gastric cancer rats	signal ↑	Food intake↑	Terawaki et al. (38)
**Years**	**Clinical research**	**Ghrelin**	**Index**	**References**
2011	Cisplatin-treated patients with gastric cancer	↗	Food consumption↑	Ohno et al. (39)
2011	Cancer cachexia patients	**↑**	Survival↑	Fujitsuka et al. (13)
2013	Gastric cancer	**↑**	Food consumption, DAUGS score↑	Takiguchi et al. (40)
2013	Gastrectomy	**→**	Body weight, GSRS↑	Gunji et al. (41)
2017	Uterine cervical or corpus cancer patients	**→**	CINV↓	Ohnishi et al. (42)
2017	Lung cancer patients with chemotherapy	**-**	CINV →	Harada et al. (43)
2019	Cisplatin-treated patients with esophageal cancer	**↑**	Food consumption↑	Hamai et al. (45)
2020	Cisplatin-treated patients with lung cancer	**↑**	Food consumption↑	Yoshiya et al. (44)
2020	Pancreaticoduodenectomy	**→**	Delayed gastric emptying →	Yamaguchi et al. (46)
**Related to stress**
**Years**	**Basic research**	**Ghrelin**	**Index**	**References**
2011	Urocortin 1-treated rats	↑	Food intake ↑	Yakabi et al. (47)
2011	Novelty stressed mice	↑	Food intake ↑	Saegusa et al. (29)
2013	Novelty stressed mice	↑	Food intake ↑	Yamada et al. (30)
2014	Urocortin 1-treated rats	↑	Food intake ↑	Yakabi et al. (32)
2014	Acute restrained stressed mice	signal ↑	Gastric motility↑	Nahata et al. (48)
2015	Urocortin 1-treated rats	↑	Gastric emptying↑	Harada et al. (49)
2020	Novelty stressed mice	-	Food intake ↑	Yamada et al. (50)
**Years**	**Clinical research**	**Ghrelin**	**Index**	**References**
2011	Esophageal cancer patients with chemotherapy	-	Nausea ↓	Seike et al. (51)
2014	Non-erosive reflux disease	-	MCS score↑	Tominaga et al. (2)

*CINV, chemotherapy-induced nausea and vomiting; DAUGS, Dysfunction After Upper Gastrointestinal Surgery for Cancer; GSRS, Gastrointestinal Symptom Rating Scale; MCS, mental component summary*.

#### Clinical Study

In gastric cancer patients (39–41) and uterine cervical or corpus cancer patients (42), the efficacy of rikkunshito for ghrelin concentration and gastrointestinal dysfunction, including feeding, was evaluated. Ohno et al. reported that rikkunshito at a dose of 7.5 g/day suppressed the increase in oral intake and the decrease in acylated ghrelin due to cisplatin administration from the start of the combined administration of S-1 and cisplatin to patients with gastric cancer (39). Similarly, Takiguchi et al. (40) observed an increase in the acylated ghrelin ratio to total ghrelin at 4 weeks after administration of rikkunshito and an improvement in the Dysfunction after Upper Gastrointestinal Surgery for Cancer (DAUGS) and visual analog scale. These results clearly suggest that rikkunshito clinically promoted ghrelin secretion and suppressed feeding-related decline. However, in patients with proximal gastrectomy, weight gain and increase in the Gastrointestinal Symptom Rating Scale (GSRS) were noticed after administering rikkunshito. Still, they did not affect ghrelin levels (41). These findings suggest that rikkunshito improves gastrointestinal symptoms in patients with gastrectomy, but its effect on ghrelin has various results. This may be mediated by the fact that the main production site of ghrelin is the stomach. The conditions for collecting blood samples and the conditions for measuring acylated ghrelin may differ at each facility. Ohnishi et al. (42) found that rikkunshito (7.5 g/day) administration for 2 weeks in patients with cervical cancer improved appetite up to 2–6 days after paclitaxel administration and delayed onset 24–120 h later, and it significantly suppressed nausea and vomiting. However, acylated ghrelin did not change even after administration of an anticancer drug, and no effect was found by rikkunshito. Moreover, it was reported that there was no effect on nausea and vomiting by chemotherapy for lung cancer patients in the group taking rikkunshito 7.5 g/day for 7 days at the same time (43) but for anorexia. The 14-day administration of rikkunshito (7.5 g/day) inhibited the decrease in plasma acylated ghrelin, and the rate of decrease in calorie intake was lower in rikkunshito than in the control course (18 vs. 25%, *P* = 0.025) (44). Another researcher reported that the median rate of reduction in food intake was significantly lower with rikkunshito than without it (2 vs. 30%; *P* = 0.02) (45). Median acylated ghrelin increased significantly from day 3 to day 8 in patients on both courses with and without rikkunshito (9.6–15.7 fmol/mL, P < 0.0001; control, 10.2–17.8 fmol/mL, *P* = 0.0002). The rate of median increase in plasma acylated ghrelin levels between days 3 and 8 tended to be higher in the rikkunshito than in the control course (68 vs. 48%, *P* = 0.08). For delayed gastric emptying after pancreaticoduodenectomy, the 21-day administration of rikkunshito showed a significantly upregulating in total ghrelin and acylated ghrelin levels compared to preoperative, but no obvious effect on delayed gastric emptying was observed (46). It is believed that the effectiveness of rikkunshito will become clearer in future large-scale studies.

### Stress-Induced Loss of Appetite

Stress is closely associated with appetite and exhibits an entirely different phenotype depending on the quality and duration of stress. Acute stress and stress loads that have a strong impact may primarily suppress feeding (52). In addition, a combination with chronic and mild stress may increase appetite and alter food preferences (53, 54). Previously, it has been reported that abnormal dynamics in acylated ghrelin are observed with stress loading and mediate abnormality in appetite. This review describes the effect of rikkunshito on the stress-induced loss of appetite.

#### Animal Model

Blood adrenocorticotropic hormone (ACTH) and corticosterone on the HPA axis in the rodent model are used as indicators of the degree of an acute stress response. Previous studies have not investigated in detail whether rikkunshito inhibits the HPA axis. It was confirmed that the administration of rikkunshito to stress-loaded aged mice significantly decreased the increase in ACTH or corticosterone value (55). This indicates that rikkunshito may act in a suppressive manner on stress itself.

Yakabi et al. (32, 47) and Harada (49) demonstrated that the ICV administration of urocortin 1, which has a strong affinity for the CRF receptor, significantly reduces feeding behavior and abnormal movement of the upper gastrointestinal tract. The concentration of acylated ghrelin in the peripheral blood was significantly reduced, simultaneously, and the supplementation of acylated ghrelin to the urocortin-treated rat significantly improved this decrease in food intake. Urocortin-induced reduction of plasma ghrelin and food intake were restored by CRF2 receptor antagonist. Administration of rikkunshito to urocortin-treated rats significantly improved reduced food intake, abnormal gastrointestinal motility, and acylated ghrelin levels (32, 47, 49). The adrenergic α2 receptor was activated in urocortin-administered rats, and it was also found that rikkunshito contained a component with an antagonistic effect on the receptor (32, 49). However, the administration of an adrenergic β1 receptor agonist also improves urocortin-induced ghrelin lowering, but it is not confirmed whether or not rikkunshito contains a β1 agonist-like ingredients component.

Rodents are often bred and managed with 3–5 animals depending on the cage size. Acute stress can be induced by acclimating to this environment for about a week and then transferring to a completely new cage and bedding (28–30, 50). Novel environmental changes can cause mild and transient corticosterone increase and decreased feeding in mice. Additionally, the concentration of acylated ghrelin in the blood decreases at the same time as stress loading (28–30). Rikkunshito significantly reduced the decrease in food intake and reduction in blood acylated ghrelin concentration due to this novel environmental change stress (28–30). Since the decreased food intake and ghrelin secretion in this model are partially canceled by the administration of 5-HT_2B_R or 5-HT_2C_R antagonists, serotonin is involved in the decreased food intake in the brain and digestive organs (28–30). ICV administration of CRF1R antagonists mediates reductions in food intake and plasma acylated ghrelin secretion (29), suggesting that intracerebral CRF1R activation is the trigger for the onset of this model. Restraint stress is known as classical physical and mental stress. Restraint stress in mice causes dysfunction of the upper gastrointestinal tract motility and a decrease in acylated/des-acyl ghrelin ratio (48). Administration of rikkunshito to stress mice significantly improved gastric motor function abnormalities such as delayed gastric emptying and gastric motility index. It is speculated that these action by rikkunshito may have canceled the decreased feeding and ghrelin secretion deficiency due to stress.

#### Clinical Study

There are few clinical evidences focusing on the efficacy of rikkunshito on stress and mental illness. Tominaga et al. found that taking rikkunshito (7.5 g/day) for 8 weeks in patients with proton pump inhibitor-resistant non-erosive reflux disease (NERD) represents the mental quality of life in patients with a low body mass index. It proved that the mental component summary (MCS) scores of the SF-8 was significantly improved by rikkunshito (2). Additionally, administration of docetaxel/5-FU/CDDP in patients with advanced esophageal cancer for 2 weeks with rikkunshito (7.5 g/day) significantly improved nausea, as well as sleep, mood, volition, daily living activity, and anxiety and greatly improved quality of life scores, including the feeling of anxiety (51). Although the results of these clinical trials do not show the direct anti-stress effect of rikkunshito, it led to the implementation of larger clinical trials and it is expected to find the usefulness of rikkunshito for gastrointestinal disorders and neuropsychiatric parameters due to stress loading.

### Mechanism of Action of Rikkunshito

#### Antagonists on Serotonin, CRF, and Adrenergic Receptors

Ghrelin release in the stomach and hypothalamus is negatively regulated by 5-HT_2B_R and _2C_R activation (11). Heptamethoxyflavone, hesperetin, nobiletin, tangeretin, and isoliquiritigenin, which are components of rikkunshito, have an antagonistic activity against 5-HT_2B_R and _2C_R *in vitro*. Additionally, these components, when administered alone *in vivo*, suppressed a decrease in blood ghrelin levels (11). Isoliquiritigenin has been confirmed to transfer to the brain after the administration of rikkunshito and may mediate the decrease in ghrelin secretion due to antagonism of 5-HT_2C_R localized in the central nervous system (56).

It is suggested that stress-related hypophagia involves abnormalities in ghrelin kinetics mediated by CRF1 and adrenergic receptors, and stimulation of CRF1 and α receptors negatively regulates ghrelin secretion. Nobiletin and isoliquiritigenin antagonize the CRF1 receptor at IC50 values of 0.36 and 0.67 μmol/L, respectively (56). In addition, glycycoumarin has an IC50 value of 5–39 μmol/L for all subtypes (_A,B,C_) of the α_2_-adrenergic receptor (AR). The IC50 value of 6-shogaol, which is a component of *Zingiberis rhizoma*, is 25 μmol/L for α_2A_-AR, 8-shogaol is 5–6 μmol/L for α_2A_, α_2C_-AR, and 10-gingerol is has an IC50 value of 5–31 μmol/L for α_2A_, α_2B_, α_2C_-AR (32).

#### Ghrelin Receptor Stimulating Effect

Previous findings have shown that rikkunshito promotes acylated ghrelin secretion, but it does not increase it beyond physiological secretion. Therefore, it was questioned whether this degree of action could improve the reduced food intake. Rikkunshito enhanced the binding to ghrelin in cells expressing GHS-R1a *in vitro* and further significantly increased [Ca^2+^] influx in the area under the curve by ghrelin. At the same time, it was discovered that the action was caused by the ingredients of rikkunshito, i.e., atractylodin (13). This finding means a new mechanism in which rikkunshito not only increases the blood acylated ghrelin concentration but also enhances the reactivity of acylated ghrelin with GHS-R1a, thereby increasing the ghrelin signal.

Another stimulating effect of the ghrelin signal has been studied and proposed. Rikkunshito mediates the production of cAMP through the inhibition of phosphodiesterase III against the adenylate cyclase-cAMP-PKA system involved in the ghrelin signal inhibitory effect of leptin via the PI3K-PDE pathway and the activation of ghrelin receptors (57). Further detailed research is required to determine how much this effect of rikkunshito affects the ghrelin signal.

## New Possibilities

### Aging, Gender Difference, and Healthy Life Expectancy

Maintaining the diet of the elderly is an important issue as a strategy to prevent sarcopenia in the global aging society. Additionally, aged people tend to lose their appetite due to changes in taste and decreased calorie consumption due to lack of exercise. Additionally, aged people often have many diseases, and maintaining the so-called healthy life expectancy and maintaining good quality of life are of utmost importance. In rodent studies, a flattening of ghrelin levels was observed in aged mice, with clearly reduced fasting ghrelin secretion compared to younger mice and, conversely, increased ghrelin levels during satiety (57). Additionally, changes in feeding behavior were also observed. Although the meal amount and meal size of the aged mice did not change compared with those of the younger mice, the number of activities at night and the food bout size were small, and the bout numbers were many. Thus, it eats little by little over time (58). Rikkunshito restores reduced feeding in aged mice without affecting acylated ghrelin levels (57).

So far, as a clinical evaluation, the impression that rikkunshito may be more effective for females than for males has been conveyed. However, there has been no evidence of gender differences in the effects of rikkunshito. Yamada et al. (50) studied and compared the effects of rikkunshito on decreased food intake in male and female mice exposed to psychological stress. Because of comparing female and male mice for events after stress loading, there was no gender difference in HPA axis activation, but food intake decreased more continuously in female mice. Female mice have a delayed increase in ghrelin rather than males and reduced responsiveness to exogenous ghrelin. In stress-loaded mice, rikkunshito showed an obvious effect of improving feeding in female mice. The cause of anorexia in female mice is thought to be ghrelin signal transduction failure in NTS, and rikkunshito was found to improve this signal disorder. Rikkunshito was more effective in women or the elderly (65 years and older) for FSSG in NERD patients (2). However, there is still no direct evidence that the action of rikkunshito as a ghrelin enhancer mediates gender differences and efficacy in the elderly. Further elucidation of the mechanism is required in the future.

It is known that calorie restriction activates various factors, including sirtuin (SIRT), and is involved in maintaining the functional decline of different tissues with aging (59). Particularly, activation of SIRT in the hypothalamus is triggered by calorie restriction and is associated with the maintenance of energy balance homeostasis (60–62). Ghrelin may increase the orexigenic signal and may cause the same condition as calorie restriction. For example, ghrelin activates adenosine monophosphate-activated protein kinase (AMPK) (63), and SIRT is also activated by AMPK (64). Therefore, it is easy to hypothesize that rikkunshito, which promotes ghrelin secretion and receptor activation, may cause SIRT activation. Fujitsuka et al. evaluated the effect of rikkunshito on healthy life expectancy using various aging-promoting mice (65). Rikkunshito induced activation of SIRT1 in the hypothalamus *in vivo*. It was also discovered that the effect was not expressed in ghrelin KO mice. Furthermore, administration of rikkunshito to ICR mice as aging-promoting models such as Klotho-deficient mice, SAMP8 mice, and normal-aged mice showed prolonged survival. Again, focal atrophy of myocardial fiber and pericarditis was significantly decreased in mice treated with rikkunshito. This study is a basic study using mice, but we hope that a clinical review will be conducted in the future and that rikkunshito will prove the possibility of improving the QOL of the elderly.

## Summary

The promoted ghrelin secretion and ghrelin signal promotion exerted by rikkunshito may play an important role for effectiveness for (i) anorexia, nausea and vomiting due to chemotherapy, (ii) severe loss of appetite and weight loss due to cancer cachexia and stress-induced hypophagia, and (iii) hypophagia in the elderly. Evidence accumulated over the last few years shows that rikkunshito is particularly effective against the loss of appetite and gastrointestinal disorders caused by chemotherapy. Additionally, as result of basic research, rikkunshito may be involved in eating disorders and in extending healthy life expectancy in the elderly. However, based on the valuable basic research obtained so far, the evaluation of large-scale clinical trials may lead to further evidence of the usefulness of rikkunshito for the benefit of patients, moreover even Japanese Kampo medicine.

## Author Contributions

CY and TH study design, data collection and analysis, and drafting of the manuscript. TH, SO, and HT study supervision. All authors contributed to the article and approved the submitted version.

## Funding

This publication was funded by Tsumura & Co.

## Conflict of Interest

CY and TH are employees of Tsumura & Co. and get a salary from it. SO and HT receive research funds from Tsumura & Co. The authors declare that this study received funding from Tsumura & Co. The funder had the following involvement with the study: the decision to submit it for publication.

## Publisher's Note

All claims expressed in this article are solely those of the authors and do not necessarily represent those of their affiliated organizations, or those of the publisher, the editors and the reviewers. Any product that may be evaluated in this article, or claim that may be made by its manufacturer, is not guaranteed or endorsed by the publisher.
